# Supplementary material for: Association between oral microbiome and five types of respiratory infections: a two-sample Mendelian randomization study in east Asian population
Source: Front Microbiol. 2024 Apr 10;15:1392473. doi: 10.3389/fmicb.2024.1392473 (PMC11039966; doi:10.3389/fmicb.2024.1392473)
Supplement: Supplementary file 1 [file Data_Sheet_1.zip › Supplementary Materials/MR plots of saliva/Pneumonia/s__Prevotella_nigrescens_mgs_3566/forest.pdf]

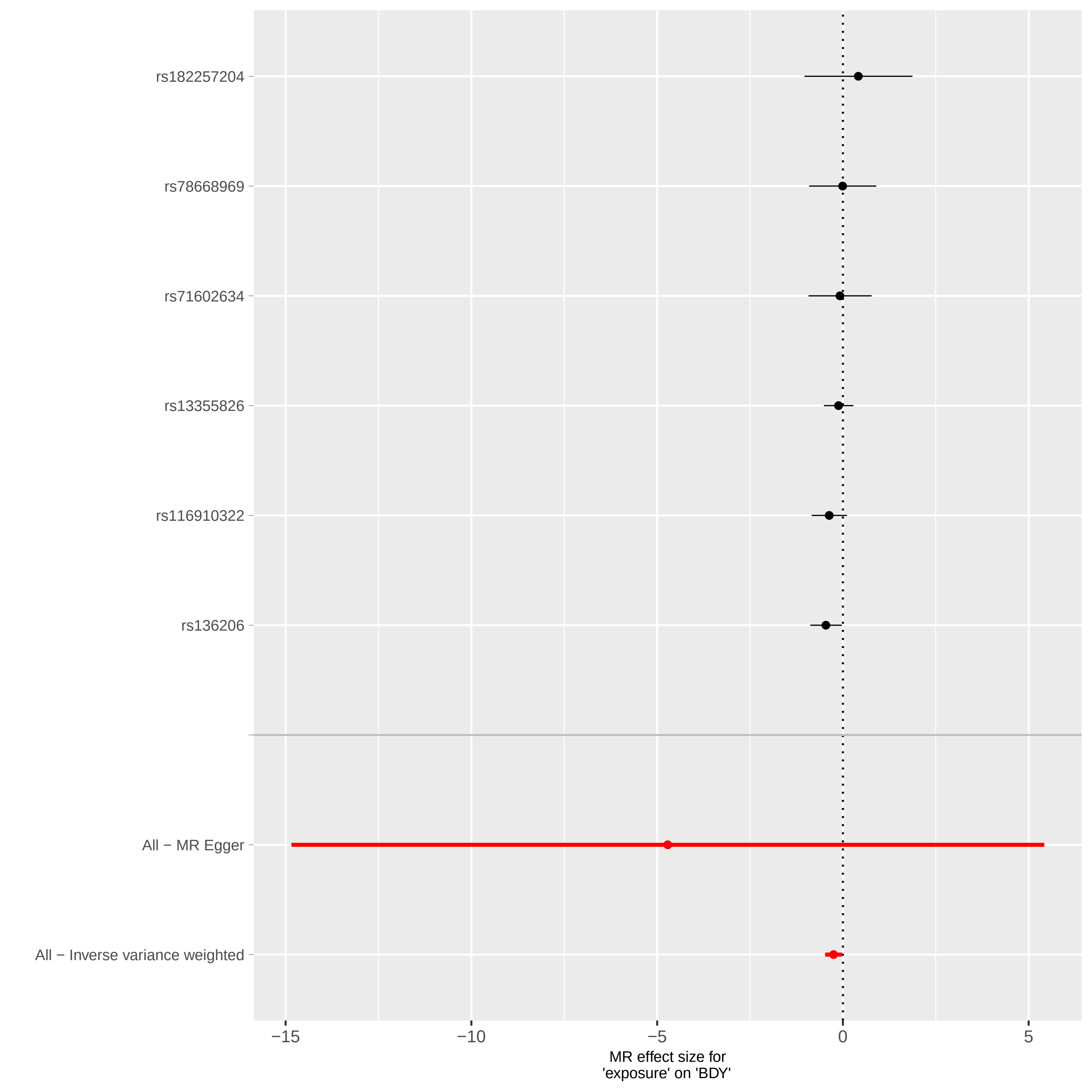

rs182257204
rs78668969
rs71602634
rs13355826
rs116910322
rs136206
All − MR Egger
All − Inverse variance weighted
−15
−10
−5
0
5
MR effect size for
'exposure' on 'BDY'
